# Fast network centrality analysis using GPUs

**DOI:** 10.1186/1471-2105-12-149

**Published:** 2011-05-12

**Authors:** Zhiao Shi, Bing Zhang

**Affiliations:** 1 Advanced Computing Center for Research & Education, Vanderbilt University, Nashville, TN 37203, USA; 2 Department of Electrical Engineering and Computer Science, Vanderbilt University, Nashville, TN 37235, USA; 3 Department of Biomedical Informatics, Vanderbilt University School of Medicine, Nashville, TN 37232, USA

## Abstract

**Background:**

With the exploding volume of data generated by continuously evolving high-throughput technologies, biological network analysis problems are growing larger in scale and craving for more computational power. General Purpose computation on Graphics Processing Units (GPGPU) provides a cost-effective technology for the study of large-scale biological networks. Designing algorithms that maximize data parallelism is the key in leveraging the power of GPUs.

**Results:**

We proposed an efficient data parallel formulation of the All-Pairs Shortest Path problem, which is the key component for shortest path-based centrality computation. A betweenness centrality algorithm built upon this formulation was developed and benchmarked against the most recent GPU-based algorithm. Speedup between 11 to 19% was observed in various simulated scale-free networks. We further designed three algorithms based on this core component to compute closeness centrality, eccentricity centrality and stress centrality. To make all these algorithms available to the research community, we developed a software package *gpu*-*fan *(GPU-based Fast Analysis of Networks) for CUDA enabled GPUs. Speedup of 10-50× compared with CPU implementations was observed for simulated scale-free networks and real world biological networks.

**Conclusions:**

*gpu*-*fan *provides a significant performance improvement for centrality computation in large-scale networks. Source code is available under the GNU Public License (GPL) at http://bioinfo.vanderbilt.edu/gpu-fan/.

## Background

Cellular systems can be modeled as networks, in which nodes are biological molecules (e.g. proteins, genes, metabolites, microRNAs, etc.) and edges are functional relationships among the molecules (e.g. protein interactions, genetic interactions, transcriptional regulations, protein modifications, metabolic reactions, etc.). In systems biology, network analysis has become an important approach for gaining insights into the massive amount of data generated by high-throughput technologies.

One of the essential tasks in network analysis is to determine the relative importance, or centrality, of the nodes based on network structure. Different centrality metrics have been proposed in the past [1]. Among them there is an important group of metrics that uses shortest path information (Table 1). Sequential implementations of the shortest path-based centrality calculation are provided in software packages such as igraph [2] and NetworkX [3]. However, these algorithms have limited applicability for large real world biological networks due to poor scalability [4]. Parallel implementations using MPI (Message Passing Interface) [4] and multi-threading [5] have been proposed to speed up graph algorithms.

**Table 1:** Shortest path-based centrality metrics

Centrality	Equation	Description
Betweenness (BC)		fraction of shortest paths between all other nodes that run through node *u*
Closeness (CC)		reciprocal of average shortest path distance
Eccentricity (EC)		reciprocal of maximum shortest path distance
Stress (SC)		total number of shortest paths between all other nodes that run through *u*

Owing to its massive parallel processing capability, General Purpose computation on Graphics Processing Units (GPGPU) provides a more efficient and cost effective alternative to conventional Central Processing Unit (CPU)-based solutions for many computationally intensive scientific applications [6]. A GPU device typically contains hundreds of processing elements or cores. These cores are grouped into a number of Streaming Multiprocessors (SM). Each core can execute a sequential thread, and the cores perform in SIMT (Single Instruction Multiple Thread) fashion where all cores in the same group execute the same instruction at the same time. NVIDIA's CUDA (Compute Unified Device Architecture) platform [7] is the most widely adopted programming model for GPU computing. In bioinformatics, GPU-based applications have already been implemented for microarray gene expression data analysis, sequence alignment and simulation of biological systems [8-11].

Parallel algorithms for centrality computation have been developed on various multi-core architectures [12-14]. However, as pointed out by Tu et al. [15], challenges such as dynamic non-contiguous memory access, unstructured parallelism, and low arithmetic density pose serious obstacles to an efficient execution on such architectures. Recently, several attempts at implementing graph algorithms, including breadth first search (BFS) and shortest path, on the CUDA platform have been reported [16-18]. Two early studies process different nodes of the same level in a network in parallel [16,17]. Specifically, for the BFS implementation, each node is mapped to a thread. The algorithms progress in levels. Each node being processed at the current level updates the costs of all its neighbors if the existing costs are higher. The algorithms stop when all the nodes are visited. This approach works well for densely connected networks. However, for scale-free biological networks [19] in which some nodes have many more neighbors than the others, these approaches can potentially be slower than implementations using only CPUs due to load imbalance for different thread blocks [18]. A recent study by Jia et al. exploits the parallelism among each node's neighbors to reduce load imbalance for different thread blocks and achieves better performance in All-Pairs Shortest Path (APSP) calculation and shortest path-based centrality analysis [18]. However, the APSP algorithm can only use one thread block per SM due to excessive memory duplication, which is an inefficient way of executing threads blocks and may result in low resource utilization [20].

In this paper, we developed a new APSP algorithm that avoids data structure duplication and thus allows scheduling units from different thread blocks to fill the long latency of expensive memory operations. We showed that our algorithm outperformed Jia's algorithm for betweenness centrality computation. Based on the improved APSP algorithm, we developed a software package *gpu*-*fan *(GPU-based Fast Analysis of Networks) for computing four widely used shortest path-based centrality metrics on CUDA enabled GPUs. Using simulated scale-free networks and real world biological networks, we demonstrated significant performance improvement for centrality computation using *gpu*-*fan *as compared to CPU implementations.

## Implementation

Given a network *G *= (*V*, *E*) with |*V*| = *n *and |*E*| = *m*, we implemented algorithms for computing four shortest path-based centrality metrics as described in Table 1 on the CUDA platform. There are currently two approaches for computing shortest paths on GPUs. The first approach processes different nodes of the same level in parallel [17]. The second one exploits the parallelism on the finest neighborhood level [18]. Since biological networks typically exhibit a scale-free property [19], the first approach can potentially cause serious load imbalance and thus result in poor performance. Therefore, we adopted the second approach in our implementation. Specifically, a network is represented with two arrays. A pair of corresponding elements from each array is an edge in the network. For undirected networks, an edge is represented by two pairs of elements, one for each direction. All four centrality metrics are based on the APSP computation. The APSP algorithm performs a BFS starting from each node. During the BFS, each edge is assigned to a thread. If one end of an edge is updating its distance value, the thread checks the other node and updates the distance value if it has not been visited yet. Each edge (thread) can proceed independently of each other and therefore exploits the finest level of parallelism to achieve load balance. After finding all shortest paths, each centrality metric is computed with additional GPU kernel function(s) as described in [18]. For betweenness centrality, the implementation is based on a fast serial version [21].

**Figure 1:**
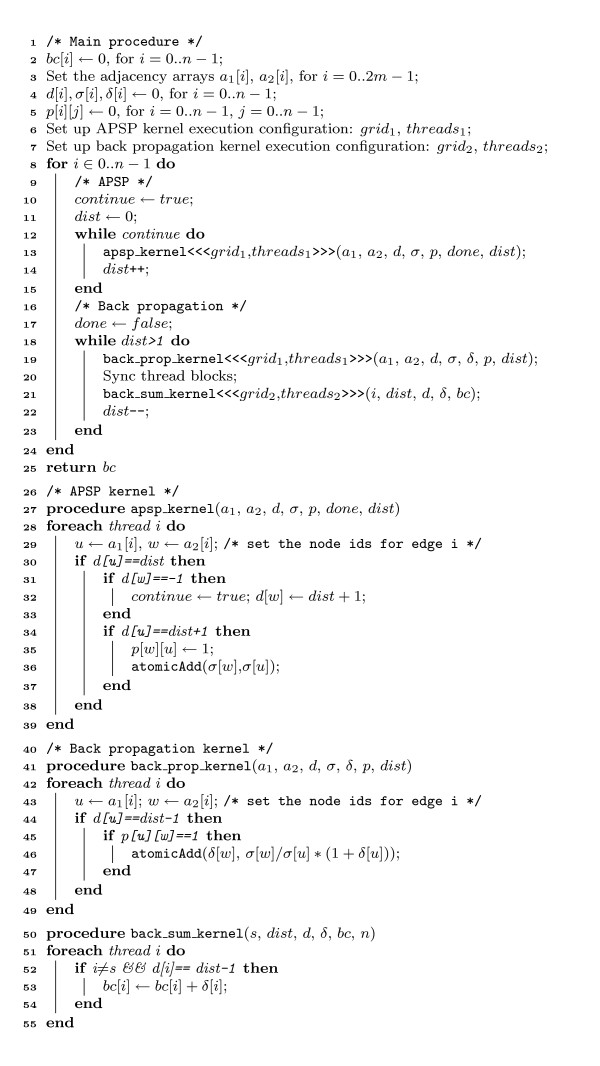
**Pseudo-code for computing betweenness centrality on GPU**. Lines 1-25 implement the main function that is executed on CPU. Code between lines 26-39 is the kernel function that carries out the All-Pairs Shortest Path algorithm. Lines 40-55 implement the back propagation where the final values of betweenness centrality for each node are set.

By design, the APSP algorithm in [18] requires duplicated allocation of several large data structures in each thread block. This effectively limits the number of thread blocks that can be launched due to limited memory size on the device. Therefore the algorithm fixes the number of thread blocks to be the number of available SMs, which is typically in the range of 10-30 for the current generation of CUDA enabled devices. Each block uses the maximum number of threads allowed for the device. In contrast, our algorithm does not duplicate the data structures and can have enough thread blocks and thus enough warps, the scheduling units on CUDA, from different thread blocks to fill the long latency of expensive memory operations. In other words, when warps from one thread block stall, warps from other thread blocks whose next instruction has its operands ready for consumption can continue. In [20], the authors proposed a metric called *utilization *to estimate the utilization of the compute resources on the GPU. The definition of the metric indicates that assigning a larger number of thread blocks on each SM without violating local resource usage can result in higher resource utilization. We let the number of threads per block vary between 64 and 512. The best overall performance was obtained with 256 threads per block (8 warps). Setting the number of threads per block to 256 allows a total of *l*/256 blocks when the APSP kernel launches, where *l *is the length of the adjacency arrays. For undirected networks studied in this work, *l *is twice the number of edges. Another advantage of setting the number of blocks in this way is that for graphs with larger number of edges, a proportionally larger number of blocks will be deployed to proactively hide the potentially high memory access latency. Figure 1 lists the pseudo-code of the betweenness centrality algorithm that uses the improved APSP kernel, where *n *and *m *are the numbers of nodes and edges in the network, respectively.

## Results and Discussions

We tested both GPU and CPU implementations on a Linux server. The server contains 2 Intel Xeon L5630 processors at 2.13 GHz, each having 4 processing cores, and an NVIDIA Tesla C2050 GPU card (448 CUDA cores, 3GB device memory). The CPU implementation was single threaded and coded in C++. The kernel functions in GPU version were implemented with CUDA C extension.

**Figure 2:**
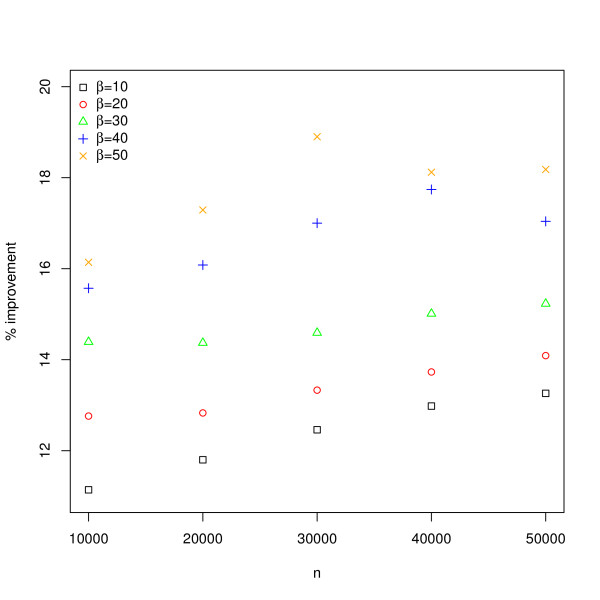
**Performance improvement over the most recent GPU-based betweenness centrality algorithm**. We benchmarked our betweenness centrality algorithm against the one described in [18]. Results are based on 25 randomly generated scale-free networks with *n *varied from 10, 000 to 50, 000 and *β *varied from 10 and 50. *n *represents the number of nodes in the network and *β *controls the preferential attachment process for generating the scale-free networks.

We first compared our algorithm with the one described in [18] for betweenness centrality calculation. Networks were generated with NetworkX based on Barabási-Albert's preferential attachment model [22]. The model has two parameters, *n *and *β*, where *n *represents the number of nodes in the network and *β *controls the preferential attachment process. Specifically, new nodes are added to the network one at a time, and each new node is connected to *β *existing nodes with a probability that is proportional to the number of edges that the existing nodes already have. We considered 25 networks with *n *varied from 10, 000 to 50, 000 and varied from 10 and 50. As shown in Figure 2, our algorithm outperformed Jia's by 11-19% for randomly generated networks owing to higher resource utilization.

**Figure 3:**
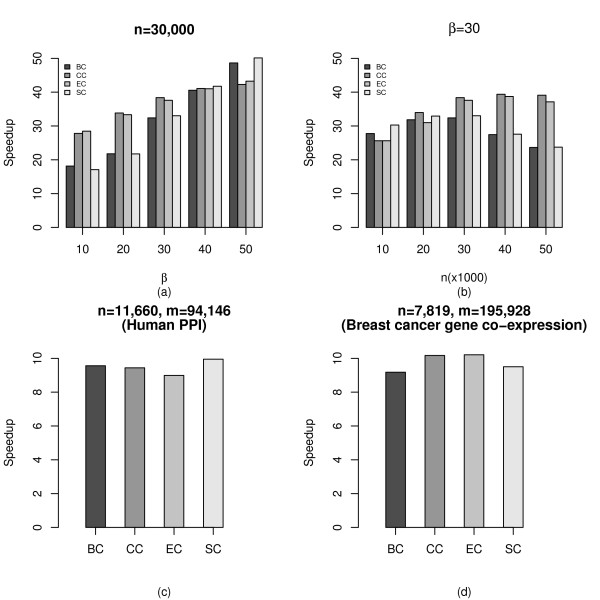
**Speedup of centrality computation with GPU as compared to CPU implementations**. (a) Speedup as a function of *β *when network size is fixed. (b) Speedup as a function of network size *n *when *β *is fixed. (c) Speedup of four centrality metrics for a human protein-protein interaction network. (d) Speedup of four centrality metrics for a breast cancer gene co-expression network. *n *represents the number of nodes in the network and *β *controls the preferential attachment process for generating the scale-free networks. BC: betweenness centrality; CC: closeness centrality; EC: eccentricity centrality; SC: stress centrality.

Based on the improved APSP algorithm, we developed a software package *gpu*-*fan *(GPU-based Fast Analysis of Networks) for computing four shortest path-based centrality metrics and then compared the performance with corresponding CPU implementations. Overall, a speedup of 11-56× over CPU implementations was observed for the aforementioned networks. Figures 3(a) and 3(b) depict representative results for the fixed *n *of 30, 000 and fixed *β *of 30. When *n *is fixed, networks with larger *β *exhibited higher speedup due to increased available data parallelism and arithmetic operations. When *β *was fixed, larger network size led to more arithmetic operations but not necessarily increased data parallelism. As a result, the speedup levels were more stable across different network sizes. The running times for a randomly generated scale-free network with *n *= 30, 000 and *β *= 50 are given in Table 2.

**Table 2:** Running times on GPU vs. CPU for centrality computations in a randomly generated scale-free network (*n *= 30, 000, *β *= 50)

Centrality	CPU time (*sec*)	GPU time (*sec*)	Speedup
Betweenness (BC)	17777.0	365.5	48.64
Closeness (CC)	3914.7	92.6	42.29
Eccentricity (EC)	3954.1	91.4	43.24
Stress (SC)	16950.1	338.2	50.12

Finally, we tested *gpu*-*fan *on a human protein-protein interaction (PPI) network and a breast cancer gene co-expression network [23]. The human PPI has 11, 660 nodes and 94, 146 edges, while the co-expression network has 7, 819 nodes and 195, 928 edges. Although these two networks have relatively low edge density, we still obtained a speedup of around 10× as shown in Figures 3(c) and 3(d).

For the computation of betweenness centrality, a two dimensional array *p *of size *n *× *n *is used to keep predecessor information, where *p*(*i*, *j*) = 1 indicates that there is a shortest path passing from node *i *to node *j*. This limits our implementation from processing graph with large number of nodes because of the limited global memory size on GPU. Since this array will likely be sparse, using sparse matrix representation can help reduce memory usage. As a future work, we will investigate the use of sparse matrix and its potential effect on the overall performance.

## Conclusions

We developed a software package for computing several shortest path-based centrality metrics on GPUs using the CUDA framework. The algorithms deliver significant speedup for both simulated scale-free networks and real life biological networks.

## Availability and requirements

**Project name**: gpu-fan (GPU-based Fast Analysis of Networks)

**Project home page**: http://bioinfo.vanderbilt.edu/gpu-fan/

**Operating system**: Unix/Linux

**Programming language**: CUDA, C/C++

**Other requirements**: CUDA Toolkit 3.0 or higher, GPU card with compute capability 2.0 or higher

**License**: GPL v3

## Abbreviations

GPGPU: General Purpose computation on Graphics Processing Units; CUDA: Compute Unified Device Architecture; GPU: Graphics Processing Unit; SIMT: Single Instruction Multiple Thread; SM: Streaming Multiprocessor; APSP: All-Pairs Shortest Path; BFS: Breadth First Search.

## Authors' contributions

BZ and ZS conceived of the study and designed the algorithms. ZS implemented the algorithms and conducted the performance analysis. Both authors drafted the manuscript and approved the final version.
